# Effects of physical exercise programs on cognitive function in Parkinson’s disease patients: A systematic review of randomized controlled trials of the last 10 years

**DOI:** 10.1371/journal.pone.0193113

**Published:** 2018-02-27

**Authors:** Franciele Cascaes da Silva, Rodrigo da Rosa Iop, Laiana Cândido de Oliveira, Alice Mathea Boll, José Gustavo Souza de Alvarenga, Paulo José Barbosa Gutierres Filho, Lídia Mara Aguiar Bezerra de Melo, André Junqueira Xavier, Rudney da Silva

**Affiliations:** 1 University of State of Santa Catarina, Center for Health Sciences and Sports, Adapted Physical Activity Laboratory, Florianópolis, Santa Catarina, Brazil; 2 University of Brasilia, Faculty of Physical Education, Brasilia, Brazil; 3 University of Southern Santa Catarina, Medicine Course, Florianópolis, Santa Catarina, Brazil; Nathan S Kline Institute, UNITED STATES

## Abstract

**Background:**

Given the relative importance of cognitive impairment, there was considerable interest in identifying the cognitive profile of PD patients, in order to ensure specific and appropriate therapeutic interventions.

**Purpose:**

To determine the effects of physical exercise programs on cognitive function in PD patients, compared with the control group.

**Data sources:**

Medline, Cochrane, Scopus, PEDro and Web of Science (last searched in September 2016).

**Study selection:**

Randomized clinical trials examining the effects of physical exercise programs and cognitive function in PD patients. Nine studies fulfilled the selection criteria and were included in this review.

**Data extraction:**

Characteristics of the publication, characteristics of the participants, test used for cognitive screening, cognitive domain assessed, tools used to assess cognitive function, characteristics of the experimental intervention, characteristics of the control group, mean results and standard deviation of function cognitive. The PEDro score was used to evaluate methodological quality.

**Data synthesis:**

Most eligible studies showed good methodological quality based on the PEDro scale. Studies have shown that adapted tango for PD patients, cognitive training combined with motor training, and treadmill training promote the preservation or improvement of cognitive function in PD patients.

**Limitations:**

The diversity of cognitive tests used to assess cognitive function and the high heterogeneity identified between the physical exercise programs.

**Conclusions:**

Physical exercise programs promote positive and significant effects on global cognitive function, processing speed, sustained attention and mental flexibility in PD patients, at a mild to moderate stage for patients with a 6-year clinical diagnosis of PD. However, treadmill training performed 3 times a week for about 60 minutes and for a period of 24 weeks produced larger improvements in cognition.

## Introduction

Parkinson’s disease (PD) is a chronic, progressive and multisystem neurodegenerative disease [1] and has historically been considered specifically as a motor disorder. [2] However, in the past decades, a wide spectrum of non-motor manifestations of this disease has been recognized. [3,4]

In this context, cognitive impairment is one of the most prominent features of PD, [5,6] considering that 25 to 30% of the patients have cognitive deficits at the onset of the disease and 50% of the patients demonstrate significant cognitive decline in the first three to five years of illness. [7,8] Mild cognitive impairment (MCI) affects between 18.9 and 38.2% of patients in the early stages of the disease. [9]

A prospective cohort study with PD patients shows that mild cognitive impairment (MCI) in the first year of PD diagnosis indicates a high risk of dementia. More than 25% of MCI patients diagnosed with PD developed dementia within 3 years of follow-up compared to less than 1% of MCI patients without a diagnosis of PD. Among MCI patients at the onset of the disease and after one year of follow-up, almost half of them evolved into dementia. [10]

The clinical status of PD has been compared to an iceberg: the visible part represents the motor symptoms, and most non-visible part represents the various non-motor manifestations, which are not often recognized in clinical practice. [11] Also, PD diagnosis is often complex and there are no reliable diagnostic biomarkers. Thus, the diagnosis is based on clinical skills and methods. [5] However, mild cognitive impairment (MCI) may even precede motor symptoms. [12]

One of the most feared PD complications for patients and their caregivers is the development of dementia. [13] The prevalence of dementia is higher than 75% in patients who survive 10 years with the diagnosis of PD. [14] It is estimated that 80% of PD patients will progress to dementia associated with PD.^14^ The term “Parkinson´s disease dementia” (PDD) refers to dementia that develops at least 12 months after the onset of the motor changes. When dementia is developed in the first 12 months of disease progression, the criteria for the diagnosis of Lewy bodies dementia are met. [15] PDD is the most severe manifestation among the cognitive changes and it increases the risk of death. [16]

In a systematic review, the prevalence of PDD was estimated at 0.5% in individuals aged 65 years or older. The percentage of PDD among individuals with dementia was 3.6% (3.1–4.1), with an estimated prevalence of PDD of 0.2% in individuals aged 65 years or older. Despite the methodological variation of the included studies, the authors suggest that 24 to 31% of PD patients show dementia and that 3 to 4% of dementia in the population could be associated with PD. The estimated prevalence of PDD in the general population over 65 years is 0.2 to 0.5%. [17]

Cognitive deficits sometimes occur in the early stages of PD, and in these cases, they may not be clinically apparent, but detectable only by specific tests. [18] In the systematic review conducted by Romann et al. [19], the authors assessed the most commonly used instruments for cognitive evaluation in PD patients undergoing deep brain stimulation and found that there is no consensus about such cognitive instruments. In another systematic review, [20] the authors demonstrated that further research is needed on cognitive function screening tools that can be used to detect early signs of cognitive impairment. Several tools do not cover all fundamental cognitive domains; moreover, there are only a few longitudinal studies with follow-up periods long enough to observe cognitive changes.

Due to the strong impact of cognitive disorders on the quality of life of patients and their caregivers, it is important to find tools needed to manage cognitive decline. [21] Given the relative importance of cognitive impairment, there was considerable interest in identifying the cognitive profile of PD patients, in order to ensure specific and appropriate therapeutic interventions. [22] From this point of view, the literature on this topic is quite comprehensive. [8,23–26]

In the systematic review conducted by Hindle et al. [27] the effectiveness of various types of interventions on cognition in PD patients was assessed. However, the study was limited to the period between 2000 and 2011, and the descriptors related to cognition were restricted. The result of the review produced four clinical studies related to physical exercise and physical therapy and four studies focusing on combined therapy. Likewise, the systematic review conducted by Murray et al. [28], which aimed to analyze the evidence on the effects of exercise on cognition in PD in humans and animal models, showed that cognition-related descriptors were also restricted, and that nine clinical studies in humans demonstrated several modalities of physical exercise.

This study aimed to verify the effects of physical exercise programs on cognitive function in Parkinson’s disease patients, compared with the control group, through a systematic review of randomized clinical trials of the last 10 years. The secondary objectives were to verify the most commonly used tools to assess cognitive screening and cognitive function, as well as to check the cognitive domains evaluated and post-intervention effects (follow-up).

## Method

This systematic review was registered under the number CRD42016047788 in the International Prospective Register of Systematic Reviews—PROSPERO and follows the recommendations proposed by the Preferred Reporting Items for Systematic Review and Meta-Analysis: The PRISMA Statement (S1 PRISMA Checklist). [29]

### Eligibility criteria

Randomized clinical trials were included in this review. All included studies investigated physical exercise programs and cognitive function in PD patients. They were indexed in previously selected databases with available abstracts, full online access of the last 10 years and no language restrictions. Such restriction has the objective to show a current panorama of the analyzed studies.

### Search strategy

MEDLINE (Medical Literature Analysis and Retrieval System on-line) via Pubmed, Cochrane, Scopus (Elsevier), PEDro and Web of Science were the selected electronic databases for this study. The search strategy included the descriptors proposed in the Medical Subject Headings (MeSH) related to the topics (see Table 1), associated with a sensitive list of search terms for randomized controlled trials (RCTs), developed by Robinson and Dickersin. [30] All search operations were performed in September 2016.

**Table 1:** Descriptors used in search strategy.

Topics	Descriptors
**Physical exercise programs**	Physical Therapy Modalities"[Mesh], "Physical Therapy Modalities", “Modalities, Physical Therapy”, “Modality, Physical Therapy”, “Physical Therapy Modality”, “Physiotherapy (Techniques)”, “Physiotherapies (Techniques)”, “Physical Therapy Techniques”, “Physical Therapy Technique”, “Techniques, Physical Therapy”, "Exercise Movement Techniques"[Mesh], "Exercise Movement Techniques", “Movement Techniques, Exercise”, “Exercise Movement Technics”, "Exercise Therapy"[Mesh], "Exercise Therapy", “Therapy, Exercise”, “Exercise Therapies”, “Therapies, Exercise”, "Exercise"[Mesh], "Exercise", “Exercises”, “Exercise, Physical”, “Exercises, Physical”, “Physical Exercise”, “Physical Exercises”, “Exercise, Isometric”, “Exercises, Isometric”, “Isometric Exercises”, “Isometric Exercise”, “Exercise, Aerobic”, “Aerobic Exercises”, “Exercises, Aerobic”, “Aerobic Exercise”, "Resistance Training"[Mesh], "Resistance Training", “Training, Resistance”, “Strength Training”, “Training, Strength”, “Weight-Lifting Strengthening Program”, “Strengthening Program, Weight-Lifting”, “Strengthening Programs, Weight-Lifting”, “Weight Lifting Strengthening Program”, “Weight-Lifting Strengthening Programs”, “Weight-Lifting Exercise Program”, “Exercise Program, Weight-Lifting”, “Exercise Programs, Weight-Lifting”, “Weight Lifting Exercise Program”, “Weight-Lifting Exercise Programs”, “Weight-Bearing Strengthening Program”, “Strengthening Program, Weight-Bearing”, “Strengthening Programs, Weight-Bearing”, “Weight Bearing Strengthening Program”, “Weight-Bearing Strengthening Programs”, “Weight-Bearing Exercise Program”, “Exercise Program, Weight-Bearing”, “Exercise Programs, Weight-Bearing”, “Weight Bearing Exercise Program”, “Weight-Bearing Exercise Programs”
**Cognitive function**	"Cognition"[Mesh], “Cognitions”, “Cognitive Function”, “Cognitive Functions”, “Function, Cognitive”, “Functions, Cognitive”, "Executive Function"[Mesh], “Executive Functions”, “Function, Executive”, “Functions, Executive”, “Executive Control”, “Executive Controls”, "Problem Solving"[Mesh], "Perception"[Mesh], “Perceptions", "Memory"[Mesh], "Attention"[Mesh], “Attentions”, “Concentration”, “Concentrations”, "Learning"[Mesh]
**Population**	"Parkinson Disease"[Mesh], "Parkinson Disease", “Idiopathic Parkinson’s Disease”, “Lewy Body Parkinson Disease”, “Lewy Body Parkinson’s Disease”, “Primary Parkinsonism”, “Parkinsonism, Primary”, “Parkinson Disease, Idiopathic”, “Parkinson’s Disease”, “Parkinson’s Disease, Idiopathic”, “Parkinson’s Disease, Lewy Body”, “Idiopathic Parkinson Disease”, “Paralysis Agitans”

Source: Author’s own production

EndNote 3.4 was used to manage reference material during searches.

### Selection of studies and data extraction

Two independent reviewers initially evaluated the studies according to titles and abstracts, identified by the search strategy. Then, the reviewers evaluated the complete articles and selected studies according to the specified eligibility criteria. Disagreements between the reviewers were resolved by consensus.

The following data was extracted from the selected studies: identification of the publication, location (country) of the study, sample size, characteristics of the participants (gender, mean age, years of education, stage of the disease and mean duration of disease), test used for cognitive screening, cognitive domain assessed, tools used to assess cognitive function, characteristics of the experimental intervention, characteristics of the control group, duration of interventions, duration of follow-up, mean results and standard deviation of function cognitive, the information about the professional who supervised the physical exercises.

### Effect size calculation

The effect size calculation was performed according to Cohen’s d, where d = M1—M2/s (mean difference between, M1—M2, divided by the standard deviation, s, in each group).

The guidelines to interpret effect sizes are given in points and classified as small, medium or large effect sizes (i.e., d = 0.2 small, d = 0.5 medium, d = 0.8 large). The effect size analysis included the studies grouped by the same assessment tool of cognitive function. Studies that did not observe significant differences for the variable of interest (function cognitive) were removed from the effect size analysis.

### Quality assessment

Two reviewers (FCS and RRI) independently assessed the methodological quality of randomized controlled trials (RCTs) using the PEDro scale, which is based on the Delphi list, developed by Verhagen and colleagues. [31] The PEDro score ranges from 3 to 9 points. We used a cut-off of 4 points on the PEDro scale to indicate good quality (equal to or greater than 5 points) and poor quality (equal to or greater than 4 points) studies. Disagreements were resolved by discussion between the reviewers.

## Results

### Literature search

**Fig 1:**
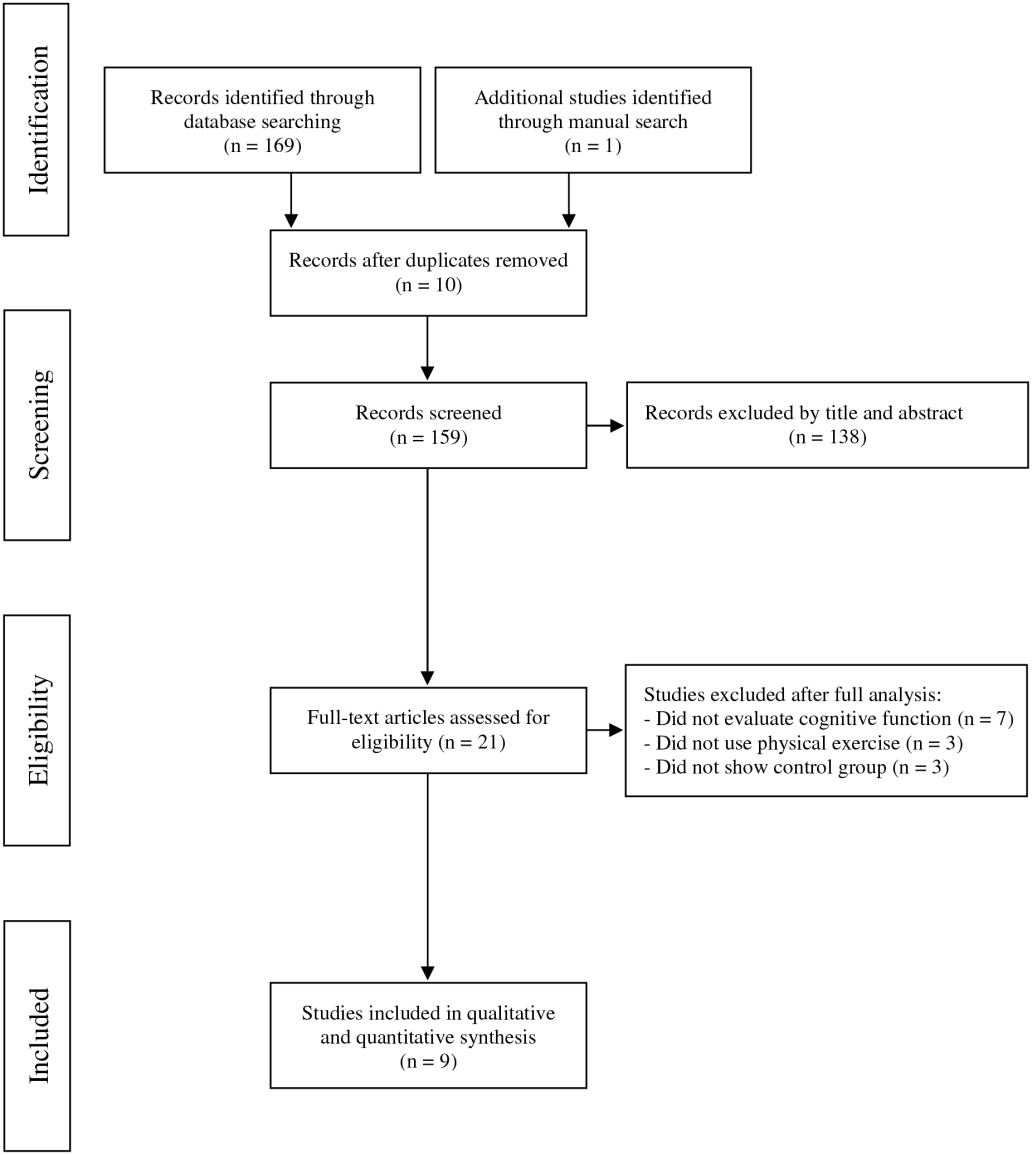
Flow diagram of 09 studies included in this review.

The literature search resulted in the identification of 169 articles. Ten studies were excluded after checking for duplicate studies, and 138 studies were excluded because their titles and abstracts did not address the theme of the present study. One article was included as a result of the manual search. Our detailed review revealed that nine studies were potentially relevant, and therefore, these studies were included. The flowchart summarizes the search strategy (Fig 1).

### Study characteristics

The main characteristics of the included studies are described in Table 2. Three studies were conducted in Canada, [32–34] two studies in the United States of America, [35,36] two studies in Brazil, [37,38] one study in Italy [39] and one in Australia. [40] The sample size ranged from 17 [39] to 39 participants. [32] Most patients were male (n = 151). The mean age of the participants in the intervention groups ranged from 59 [32] to 71.2 years old [39] and in the control groups, it ranged from 60.6 [40] to 74.4 years old. [35] The stage of Parkinson’s disease (Hoehn and Yahr) varied from mild to moderate, and the mean duration of the disease ranged from 4.7 [37] to 11.2 years. [39]

**Table 2:** Main characteristics of the studies included in this review.

*First Author, year*	*Location of the study (country)*	*Sample (n)*	*Gender*	*Mean age (years)*	*Years of Education*	*Stage of the disease (Hoehn and Yahr)*	*Mean duration of the disease (years)*	*Cognitive screening*	*Cognitive domain*	*Tool used to assess cognitive function*
Picelli, 2016 [39]	Italy	17	Intervention group–treadmill: M = 5/F = 4 Control: M = 4/F = 4	Intervention group–treadmill: 71.2 Control: 71.6	NR	Mild to moderate	Intervention group–treadmill: 11.2 Control: 10.8	MMSE > 24	Executive function, global cognitive function, processing speed, sustained attention, cognitive flexibility, operational memory	FAB-it; MoCA; Trail Making Test A and B; MI test
Rios Romenets, 2015 [34]	Canada	33	Tango: M = 12/F = 6 Control: M = 7/F = 8	Tango: 63.2 Control: 64.3	NR	Tango = 2 Control = 1.7	Tango = 5.5 Control = 7.7	Movement Disorder Study—criteria for the clinical diagnosis of Parkinon´s disease dementia (PDD)	Global Cognitive Function—Visual- spatial abilities—Attention	MoCA
Duchesne, 2015 [32]	Canada	39	PD: M = 13/F = 6 HC: M = 8/F = 12	PD: 59 HC: 64	PD: 15.05 HC: 15.7	PD: 2	PD: 8.1	MMSE and MoCA > 24	Executive function	Stroop test; Trail Making Test A and B
Nadeau, 2014 [33]	Canada	34	Control: M = 9/F = 2 Speed TT: M = 8/F = 4 Mixed TT: M = 10/F = 1	Speed TT: 64.0 Mixed TT: 60.1 Control:64.3	NR	Speed TT: 1.92 Mixed TT: 1.95 Control:1.86	NR	MMSE > 24	Global Cognitive Function Cognition	MMSE; PDQ-39
Nocera, 2013 [36]	USA	21	Tai-Chi: M = 7/F = 8 Control group: M = 4/F = 2	Tai-Chi: 66 Control: 65	Tai-Chi: 16 Control group: 15	NR	Tai-Chi: 8.08 Control group: 6.83	MMSE > 26	Cognition Attention Executive function Language Memory	PDQ-39; Trail Making Test (A-B); Stroop Color Word test; Letter Verbal Fluency; Category Verbal Fluency; Digits Span Backwards subtest of Wechsler Memory Scale
McKee, 2013 [35]	USA	33	Tango: 12M/12F Lecture Series:8M/1F	Tango: 68.45 Lecture Series: 74.4	Tango: 16.5 Lecture Series: 16.4	NR	Tango: 7.0±5.5 Lecture Series: 7.2±4.9	MoCA	Global Cognitive Function Executive function Visual- spatial function	MoCA; Brooks Spatial Task; Reverse Corsi Blocks
Pompeu, 2012 [37]	Brazil	32	M:17 F:15	Experimental group: 68.6 Control group: 66.2	5–15	1,7	Control group: 5.2±3.4 Experimental group: 4.7±5.4	MMSE > 23	Global Cognitive Function	MoCA
Cruise, 2011 [40]	Australia	28	Experimental group: M = 9/F = 6 Control group: M = 9/F = 4	Experimental group: 59.47 Control group: 60.6	NR	NR	Experimental group: 5.87±3.18 Control group: 5.46±3.63	MMSE > 24	Memory Executive function Language	Verbal Fluency (F,A,S); Spatial Working Memory (SWM); Stockings of Cambridge (SOC); Spatial Recognition Memory (SRM); Pattern Recognition Memory (PRM); Semantic Fluency for Animals
Tanaka, 2009 [38]	Brazil	20	Training group: M = 5/F = 5 Control group: M = 4/F = 6	Training group: 64.80 Control group: 64.6	NR	Training group: 1.40 Control group: 1.75	NR	MMSE	Executive function Memory	Wisconsin Card Sorting Test; Wechsler Adult Intelligence Scale III

Source: Author’s own production

In relation to the interventions developed, several forms of physical exercises can be observed, such as treadmill training, dance (Argentine Tango and Adapted Tango for PD patients), stationary bicycle training, Tai-Chi, cognitive training (Wii FitTM) combined with motor training (stretching, strengthening and exercises of axial mobility), and multimodal exercise (stretching, strengthening, exercises for coordination and balance) (Table 3). In most studies (n = 7), participants in the control group exercised without supervision, performed mild to moderate intensity physical activities, attended lectures with structured activities, and joined discussions on "physical, mental and social well-being and scientific advances related to the Parkinson’s disease”, or were instructed to maintain their usual activities. The interventions were performed two to three times a week during 40 to 90 minutes in each session. The length of the programs varied from 7 [37] to 24 weeks [38] and only two studies presented follow-up ranging from 2 [37] to 3 months. [35] Generally, the studies used moderate and high intensities controlled by calculating maximal repetition, maximal heart rate, subjective perception of exertion and VO2max.

**Table 3:** Main intervention characteristics included in this review.

*First Author, Year*	*Experimental intervention group*	*Control Group*	*Duration of interventions (weeks)*	*Follow-up (months)*	*Professionals responsible for the intervention*
Picelli, 2016 [39]	Training on treadmill. 1 session a day during 45 minutes, 3 sessions per week. The intervention consisted of 3 stages, with a 5-minute rest between each one: 10 minutes at 1.0Km / h, 10 minutes at 1.5Km/h and 10 minutes at 2.0Km/h	There was no physical training; however, they were instructed to have some type of social interaction at the same frequency and duration of the intervention group training	4	-	NR
Rios Romenets, 2015 [34]	24 Argentine tango classes as a couple, during 60 minutes, 2 sessions per week	24 sessions of physical exercises to be performed alone (specified in a booklet that was handed out to the participants)	12	-	Instructors of professional tango dancing
Duchesne, 2015 [32]	3 weekly sessions of high intensity stationary bicycle. In the beginning, the training lasted 20 minutes at 60% of VO2max. Each session added 5 minutes and 5% intensity until reaching 40 minutes of training and 80% of VO2max	3 weekly sessions of high intensity stationary cycling. In the beginning, the training lasted 20 minutes at 60% of VO2max. Each session added 5 minutes and 5% intensity until reaching 40 minutes of training and 80% of VO2max	12	-	Specialized physiotherapist
Nadeau, 2014 [33]	Treadmill training, 3 sessions per week, 60 minutes. Each participant performed 80% of the preferred speed during the first week. In the second and third weeks, all participants were encouraged to reach 90 and 100% of their walking speed, respectively. The speed was increased by 0.2 km/h in the next session after reaching 100% of its preferred speed. For the Speed TT group, the speed was increased in the following session by 0.2km/h when the participants perceived their physical exertion as moderate (4 on the Modified Borg Scale) and when the heart rate was below 75% of HRmax. For the Mixed TT group, the inclination of the treadmill was increased by 1%in the following session, when the same progression criteria based on the modified Borg scale and the heart rate were met. Next, the speed of the treadmill (0.2km/h) and the inclination of the treadmill (+ 1%) were increased alternately when the progression criteria were met.	2 sessions per week and one at home with prescribed exercises. Light and moderate intensity activities: elements of Tai-Chi, Latin dance, resistance band exercises, motor coordination movements. The intensity was adjusted according to the range of movements, duration and speed of exercise.	24	-	NR
Nocera, 2013 [36]	Tai-Chi sessions during 60 minutes, 3 sessions per week	No intervention	16	-	Tai-Chi Master with more than 20 years of experience
McKee, 2013 [35]	Adapted Tango lessons. 90-minute sessions, twice a week	90-minute lectures with structured activities and discussions on physical, mental and social well-being as well as scientific advances related to Parkinson’s Disease" given by medical students and university professors	24	3	Dance instructors who attended the Adapted Tango Workshop and with experience in working with elderly people
Pompeu, 2012 [37]	14 sessions of 60 minutes, twice a week. 30 minutes of stretching, strengthening and axial mobility exercises and 30 minutes of WiiFit^™^ exercise game	30 minutes of stretching, strengthening and axial mobility and 30 minutes of balance exercises without feedback / follow-up / assistance from professionals	7	2	Physiotherapists
Cruise, 2011 [40]	2 sessions per week during 60 minutes. The training includes strength exercises with an increase of 5–10% RM per session. The aerobic workout consisted of 25–30 minutes of cycling, rowing or treadmill at 60–85% of maximal heart rate	Maintenance of usual routine	12	-	NR
Tanaka, 2009 [38]	Multimodal exercises (flexibility—stretching; muscular resistance -specific exercises for large muscle groups; motor coordination- rhythmic activities and balance recreational motor activities). Sixty-minute sessions were held 3 times per week. The intervention consisted of 6 phases, during which there was a progressive increase of the load	Maintenance of usual routine	24	-	Physical education professionals

Source: Author’s own production

The Mini-Mental State Examination (MMSE) was the most widely used tool for cognitive screening (n = 7). [32,33,36–40] The most commonly assessed cognitive domains were: executive function (n = 6), [32,35,36,38,39,40] global cognition (n = 5), [33–35,37,39] attention (n = 3), [34,36,39] memory (n = 3), [36,38,40] language (n = 2), [36,40] and cognition (n = 2). [33,36] In order to assess cognitive function, the most used tools included the Montreal Cognitive Assessment (MoCA, n = 4), [34,35,37,39] the Trail Making Test (TMT, n = 3) [32,36,39] and the Parkinson´s Disease Questionnaire-39 (PDQ-39, n = 2). [33,36]

According to the results of mean standard deviation of the studies that showed statistically significant difference, it is generally observed that physical exercise programs promote the preservation or improvement of cognitive function in PD patients (Table 4). [33,35,37,39] Also, the effect size analysis demonstrated effects of small magnitude in global cognitive function, [35,37] processing speed, sustained attention, cognitive flexibility, [39] and cognition. [33] An effect of large magnitude was found in cognition, [33] as shown in Table 4. Furthermore, two studies showed a small magnitude effect in global cognitive function after follow-up periods of two and three months. [35,37]

**Table 4:** Results of cognitive function (assessed by MoCA, Trail Making Test and PDQ-39) of the studies included in this review in mean and standard deviation and the effect size of the studies that showed statistically significant differences.

First author, year	Experimental Intervention Group- Pre	Experimental Intervention Group- Post/ Follow-up	Intervention Control Group -Pre	Intervention Control Group- Post/ Follow-up	Effect size	Effect size Follow-up
***MoCA—Global Cognitive Function***						
**Mckee, 2013** [35]	24.9 (3.3)	26.1 (3.3)/ 26.3 (3.9)	26 (2.8)	26.3 (2.3)/ 27.4 (2.5)	0.07	0.33
**Pompeu, 2012** [37]	20.6 (4.5)	22.2 (4.5)/ 21.8 (4.5)	21.7 (4.6)	23.1 (4.6)/ 23.3 (3.4)	0.19	0.37
***Trail Making Test (A)—Processing speed and sustained attention***						
**Picelli, 2016** [39]	141.00 (113.99)	120.67 (104.59)	123.50 (101.27)	124.75 (108.55)	0.03	-
***Trail Making Test (B)—Cognitive flexibility***						
**Picelli, 2016** [39]	200.00 (80.19)	149.56 (69.33)	195.25 (93.92)	181.13 (78.96)	0.42	-
***PDQ-39—Cognition***						
**Nadeau, 2014** [33]	Speed TT: 31.3 (12.5) Mixed TT: 22.7 (14.3)	Speed TT: 40.1 (12.3) 35.4 (13.9) Mixed TT: 26.7 (14.0) 19.3 (14.1)	22.2 (16.4)	21.0 (16.6) 23.9 (17.6)	Speed TT: 1.30 Mixed TT: 0.37	-

Source: Author’s own production

### Quality assessment

Table 5 summarizes the quality of the included studies. The total scores for methodological quality ranged from 3 to 9 points: four studies had poor methodological quality [32,35,38,40] and five studies had good methodological quality. [33,34,36,37,39]

**Table 5:** PEDro scale of quality for eligible randomized controlled trials.

First Author, year	Eligibility criteria*	Random allocation	Concealed allocation	Similar at baseline	Blind subjects	Blind therapist	Blind assessors	< 15% dropout	Intention-to-treat analysis	Between-group comparison	Point measures and variability data	Total
Picelli, 2016 [39]	1	1	1	1	1	1	1	-	1	1	1	9
Rios Romenets, 2015 [34]	1	1	1	1	-	-	-	-	1	1	1	9
Duchesne, 2015 [32]	1	-	-	1	-	-	-	-	—	1	1	3
Nadeau, 2014 [33]	1	1	1	1	1	1	1	-	-	1	1	8
Nocera, 2013 [36]	1	1	-	1	-	-	1	1	-	1	1	6
McKee, 2013 [35]	1	-	-	1	-	-	1	-	-	1	1	4
Pompeu, 2012 [37]	1	1	1	1	1	1	1	-	-	1	1	8
Cruise, 2011 [40]	1	-	-	1	-	-	-	1	-	1	1	4
Tanaka, 2009 [38]	1	-	-	1	-	-	-	-	-	1	1	3

Note:

* Criterion 1 is not considered for the final score because it is an item that assesses the external validity. [41]

Source: Author’s own production

## Discussion

The primary objective of this study was to assess the effects of physical exercise programs on cognitive function in PD patients, compared to the control group, through a systematic review of randomized controlled trials (RCTs) in the last 10 years. Nine RCTs were analyzed including the effect of various types of physical exercise programs on cognitive function in PD patients in the mild to moderate stage (Hoehn and Yahr) and with disease time around 6 years.

In general, studies have shown that physical exercise programs promote the preservation or improvement of cognitive function in PD patients. The positive and significant effects of physical exercises on cognitive function in PD patients occurred specifically in the following interventions: adapted tango for PD patients, [35] cognitive training (Wii Fit^™^) combined with motor training (stretching, strengthening and axial mobility exercises), [37] and treadmill training. [33,39]

Some studies involving PD patients and elderly individuals suggest that some domains of cognitive function may improve with dance. Longer dance interventions may be needed to affect cognitive abilities that were not improved with short-term interventions, such as executive function, visual-spatial memory, or fluid intelligence. [35,42] The effects of mixed dance styles on executive function, assessed using the Frontal Assessment Battery (FAB), as well as greater benefits could be observed with dance, compared to exercise or non-intervention. Moreover, participants in mixed dance styles improved response time for a task of mental rotation. [43]

According to McNeely, Duncan and Earhart, [44] in PD, tango is the most frequently chosen dance style for interventions. [34, 35,45–47] The motor and cognitive benefits promoted by tango on motor and non-motor symptoms in PD can be explained by the sequences of movements and gestures demanded by the dance, including rhythmic back-and-forth walk, rotation, changing speeding tempo, as well as increasing self-esteem and socialization. In the study conducted by McKee and Hackney, [35] the adapted tango dancing for PD patients promoted significant improvement in global cognitive function (MoCA) after 24 weeks and after 3 months of follow-up, when compared to the control group, who attended lectures only. The cognitive benefit may also be related to the effect of aerobic exercise on cognition. [48]

Studies have reported improvement in cognitive functions in PD patients through cognitive training associated with motor training. [49] In the study conducted by Pompeu et al., [37] both groups (intervention and control) showed a significant effect on the cognitive test (MoCA) after training (14 sessions of 60 minutes, twice a week). The intervention group performed cognitive training (Wii Fit^™^) combined with motor training (stretching, strengthening and axial mobility exercises), and the control group, only motor training. These motor and cognitive improvements may be related to the physical and cognitive capacities stimulated through the gameplay. [50] According to Petzinger et al., [49] incorporating aerobic exercise specifically into cognitive training may potentiate neuroplasticity as well as improve cognitive function and motor skills.

Another intervention that showed benefits was treadmill training. Picelli et al. [39] observed a significant improvement in processing speed and sustained attention (Trail Making Test A), as well as cognitive flexibility (Trail Making Test B) after 4 weeks, compared to the control group, who maintained their usual activities. Additionally, the effect size analysis showed a large magnitude effect of treadmill training on cognition in PD patients in the mild stage (Hoehn and Yahr mean of 1.92), performing 3 sessions of 60 minutes per week, between 80 and 100% of the preferred speed, with an increase in speed of 0.2 km/h, when the participants perceived physical effort as moderate (4 on the Modified Borg Scale) and when heart rate was below 75% HRmax. [33]

A meta-analysis of RCTs (29 studies, 2,049 participants), which aimed to assess the effects of aerobic training on neurocognitive performance, showed that aerobic exercise produced modest improvements in attention, speed, executive function and memory among adults without dementia. Exercise programs ranged from 6 weeks to 18 months during 2.5 to 4 months, 3 times a week and with an intensity of 70% of maximum oxygen consumption (VO2max). The training included walking and/or running compared to the control group (waiting list or other conditions of comparison, such as stretching and strengthening, health education or relaxation). Trials that used longer interventions were associated with improved attention and processing speeds, whereas trials conducted among individuals with mild cognitive impairment (MCI) tended to show more memory improvement compared to non-dementia samples. [48]

Although it is well-established that physical exercises affect brain plasticity, different types of exercises have been found to selectively affect various regions of the brain. [51] Specifically, aerobic training has demonstrated greater benefit in the superior temporal and parietal prefrontal cortex, and the anterior and transverse tracts between the frontal and parietal lobes, i.e., in the areas involved in cognition and daily life functioning. [52]

In a large sample of adults (≥ 50 yo) with subjective memory impairment, moderate-intensity aerobic exercise performed at least during three 50-minute sessions per week promoted modest improvement in cognitive memory, language and praxis tests (Alzheimer’s Disease Assessment Scale—ADAS-Cog) after a training period of six months and a follow-up period of 18 months. The participants in the exercise group also improved delayed recall of a word list. [53]

Aerobic exercise may particularly affect executive function in PD. [49] Moderate-intensity aerobic exercise performed 2–3 times per week produced promising effects on executive function in the mild to moderate stages of PD. [54] Small preliminary case studies showed that 8 weeks of aerobic exercise, conducted 3 times a week during 20–40 minutes, produced improvements in executive function, verbal fluency and working memory in three PD patients: one with high cognitive performance (66 yo) and two with cognitive deficits (61–72 yo). [55,56]

In the systematic review conducted by Murray et al., [28] eight clinical trials in humans demonstrate that various modalities and levels of exercise intensity have also improved cognitive ability in PD, including aerobic exercise, strengthening, cognitive training (Wii Fit^™^) combined with motor and dance training. In the systematic review conducted by Cusso et al., [51] the authors assessed the effectiveness of physical activity, including physiotherapy and occupational therapy as an intervention in the non-motor symptoms of PD, namely cognition, among others. All studies used an active intervention, namely, aerobic training, treadmill training, walking, strength training, balance training, Tai-Chi, Qigong, physiotherapy, occupational therapy, Argentinean tango, Nordic walking, among others.

Two studies performed follow-up of periods of 2 [35] and 3 months. [37] The small-sized magnitude effect of adapted tango for PD patients [37] and cognitive training (Wii Fit^™^) combined with motor training (stretching, strengthening and axial mobility exercises) [35] on global cognitive function was maintained after the follow-up period. Although the improvements were maintained, it can be observed that there was not enough data to evaluate the follow-up effect, which is extremely important for clinical decision.

The Mini-Mental State Examination (MMSE) was the most widely used tool for cognitive screening. MMSE is a general screening tool for cognitive impairment. [57] It is a screening test commonly used for dementia; however, its dependence on age and educational level makes it difficult to use a rigid cut score. [58] MMSE demonstrates floor effects in individuals with severe cognitive impairment and ceiling effects in individuals with mild cognitive impairment. [59] Thus, MMSE is considered less sensitive to mild cognitive impairment in PD. [60] Its reliability for the diagnosis of dementia in Alzheimer’s disease (AD) and other dementias is not established and patients with PD and cognitive impairment may exceed the normal range of the scale. [61]

In a study involving 873 PD patients (age 70.5 ± 8.6 years, educational level 9.7 ± 2.4 years, disease duration 6.7 ± 5 years, Hoehn & Yahr 2.7 ± 1.0, MMSE score 27.3 ± 3.4), a cut-off score ≤ 24 on MMSE revealed low sensitivity for the diagnosis of Parkinson’s disease dementia (PDD). [62]

No data regarding the validation of MMSE in populations with PD has been reported. [63] Nonetheless, screening tools, such as MMSE, serve as evaluation indicators in order to provide quantitative information about global cognitive function.

The most evaluated cognitive domains were executive function, global cognitive function, attention, memory, language and cognition. It is well established that PD affects cognition. [64,65] The term "cognition" describes multiple mental processes, including executive function. Executive function includes, but is not limited to, judgment, planning, initiation, abstraction, problem solving, sequencing, and mental flexibility. [66] Executive function and visual-spatial domains may be affected after the onset of PD, [67,68] and changes in executive functions may predict the onset of dementia. [22]

According to Hanagasi, Tufekcioglu, and Emre, [69] besides the deficit in executive and visual-spatial functions, the cognitive profile of PD patients may also be characterized by attention and memory deficits. In a 5-year cohort study, approximately two-thirds of the patients showed cognitive decline. The greatest decline was observed in the areas of processing speed and memory. Elderly patients at the onset of the disease and with memory impairment at an early stage of the disease are more at risk of further cognitive decline. [7]

The most commonly used tools to assess cognitive function were the Montreal Cognitive Assessment (MoCA), the Trail Making Test A and B, and the Parkinson´s Disease Questionnaire-39 (PDQ-39). The Movement Disorder Society (MDS) diagnostic criteria for mild cognitive impairment recommend that a detailed assessment of cognitive function requires at least two tests per cognitive domain. However, MDS also stated that excessive testing per cognitive domain might cause bias. [70]

In the review conducted by Cusso et al., [51] several tests were used to assess cognition in PD patients, such as the Mini-Mental State Examination (MMSE), the Montreal Cognitive Assessment (MoCA), the subsections of the Cognitive Assessment Battery (CAB), the Stroop Test, and the Brief Test of Attention. Kalron and Zeilig [71] reviews included three clinical trials involving PD patients and the tools used to assess cognitive function were: Wisconsin Card Sorting Task, Verbal Fluency (F,A,S), Spatial Working Memory (SWM), Stockings of Cambridge (SOC), Spatial Recognition Memory (SRM), Pattern Recognition Memory (PRM), Semantic Fluency for Animals and Montreal Cognitive Assessment (MoCA).

The Montreal Cognitive Assessment (MoCA) was the most used tool in this review and it was developed as a screening tool for mild cognitive impairment (MIC). [72] MoCA is a small cognitive screening tool that is similar to the widely used MMSE, but is more sensitive to identifying MIC in the general population than MMSE. The test requires about 10 minutes to be administered in the general population and can be freely used for clinical purposes. MoCA includes tests of cognitive domains of executive and visual-spatial functions, memory, language and attention. It has adequate psychometric properties for a brief assessment of overall cognition in PD. [73]

The Trail Making Test (TMT) is another commonly used tool. It consists of two parts: A and B. In both parts, the subject is required to draw a line in the shortest possible time and without taking the pencil out of the paper. In part A, the subject is instructed to draw a line connecting the numbers 1 to 25 in ascending order. In part B, there is a greater cognitive demand on the subject´s attention, since (s)he should draw a line alternating between numbers 1 and 13 and letters A to L, i.e., 1-A, 2-B, 3-C and so on. The test assesses the aspects of sustained attention, alternating attention, mental flexibility, visual processing speed and motor function. It also evaluates visual scanning ability [74–76] It is widely used in clinical practice and research. [74] The time needed to complete the TMT is affected by age and educational level [76] and should therefore be considered in the stratification of normative samples. [74] The Trail Making Test is sensitive to the cognitive changes of Parkinson’s disease. [75]

The Parkinson´s Disease Questionnaire-39 (PDQ-39) was used in two studies. [33,36] This tool assesses the quality of life and its Brazilian version is a reliable and valid measure for PD patients in Brazil. [77] The PDQ-39 is a self-administered questionnaire with 39 items and divided into eight domains, one of which is cognition. [78]

Most eligible studies showed good methodological quality based on the PEDro scale, which suggests that the data presented are reliable. However, these results should be carefully analyzed. The diversity of cognitive tests used to assess cognitive function and the high heterogeneity identified between the physical exercise programs in the included studies made it impossible to reach a consensus on the best type of physical exercise, frequency or intensity of an intervention that may be more effective for the cognitive function of PD patients.

## Conclusion

Through this systematic review of RCTs, we found that physical exercise programs promote positive and significant effects on global cognitive function, processing speed, sustained attention and mental flexibility in Parkinson’s disease patients, at a mild to moderate stage for patients with a 6-year clinical diagnosis of PD, such as tango, cognitive training associated with motor training, and treadmill training. However, treadmill training (80–100% of the preferred walking speed) performed 3 times a week for about 60 minutes and for a period of 24 weeks produced larger improvements in cognition.

## Recommendations

It is important to consider the impact of impaired cognition on managing motor deficits associated with PD and the important contribution to the general well-being of the individual, since PD patients show an increased risk of dementia compared to the healthy population. [79,80] With cognitive impairment beginning in the early stages of PD, [81] there is a corresponding need for early cognitive strategies. [82] Medications have some effect on Parkinson´s disease dementia, but there is no convincing evidence that the progression from mild cognitive impairment (MCI) to dementia can be delayed or prevented. [83]

In this sense, this review provides a current overview of the main tools used for cognitive screening as well as the evaluation/evolution of cognitive impairment with a physical exercise program. The presence of non-motor symptoms in PD patients should be systematically researched, such as cognitive impairment, in order to contribute significantly to the structuring of physical exercise programs. Professionals who are directly involved in rehabilitation programs should understand the importance of the role of cognition in learning and motor skills, as this will assist in rehabilitation planning and management. Emerging researches suggest that aerobic exercise may improve cognition in PD. However, comprehensive and well-controlled studies should be conducted on the effects of physical exercise on cognitive function in PD patients. [82]

## Supporting information

S1 File PRISMA checklist. (DOC)
